# Cumulative Surgeon Experience and Anastomotic Leakage After Left-Sided and Segmental Colorectal Resection with Primary Anastomosis: A Seven-Year Single-Center Retrospective Study

**DOI:** 10.3390/medicina62061151

**Published:** 2026-06-13

**Authors:** Roland Sebastian Horváth, Abel Emanuel Moca, Anamaria Gozman-Pop, Árpád Rózsa, Diána Róza Pacadzisz, Teodor Andrei Maghiar, Octavian Adrian Maghiar, Paula Bianca Maghiar, Marius Adrian Maghiar

**Affiliations:** 1 Doctoral School of Biomedical Sciences, University of Oradea, 410087 Oradea, Romania; horvath_rolands@yahoo.com; 2 Békés County Central Hospital, 5700 Gyula, Hungary; drrozsaarpad@gmail.com (Á.R.); pacadzisz@gmail.com (D.R.P.); 3 Department of Dentistry, Faculty of Medicine and Pharmacy, University of Oradea, 410087 Oradea, Romania; 4 Bihor County Emergency Hospital, 410167 Oradea, Romania; anamaria.gozmanpop@gmail.com; 5 Department of Surgical Disciplines, Faculty of Medicine and Pharmacy, University of Oradea, 410087 Oradea, Romania; teodor.maghiar@yahoo.com (T.A.M.); octimaghiar@gmail.com (O.A.M.); pao.badea@gmail.com (P.B.M.); amaghiar@gmail.com (M.A.M.)

**Keywords:** anastomotic leakage, colorectal resection, surgeon experience, postoperative complications

## Abstract

*Background and Objectives:* Anastomotic leakage (AL) remains one of the most feared complications following colorectal resection, yet the relationship between cumulative surgeon experience and AL risk remains inconclusive in the literature. Most available evidence originates from high-volume or specialized centers, with limited data from mid-volume Central and Eastern European settings. This study aimed to evaluate the association between cumulative surgeon experience, operative time, and AL risk in a selected sample of colorectal resections with primary anastomosis for both benign and malignant indications, excluding right colectomies, abdominoperineal resections, TaTME, Hartmann’s procedures, and stoma-protected anastomoses, within a single-center multi-surgeon setting over a seven-year period. *Materials and Methods*: This retrospective observational study included 315 consecutive adult patients who underwent left-sided or segmental colorectal resection with primary anastomosis for both benign and malignant indications (excluding right colectomy, abdominoperineal resection, TaTME, Hartmann’s procedure, and stoma-protected cases) at Békés County Central Hospital, Gyula, Hungary, between January 2018 and December 2024. AL was defined according to ISREC criteria, with only clinically relevant grade B or C leaks recorded as events. The main exposure was cumulative surgeon experience (log2-transformed). The primary analysis used a multivariable generalized estimating equation (GEE) model clustered by surgeon, adjusted for operative time, surgical approach, conversion, wound infection, and resected segment. Eight surgeons participated, with cumulative experience ranging from 50 to 600 cases. *Results:* Among the 315 patients included, the median age was 68 years, with a male predominance (61.0%); most cases involved malignant pathology (82.9%) and at least one comorbidity (73.3%). The rectosigmoid was the most frequently resected segment (49.8%), and an open approach was used in 58.7% of cases. The overall AL incidence was 7.94% (25/315), with a median onset at postoperative day 5. In the multivariable GEE model, cumulative surgeon experience was not significantly associated with AL risk (OR per doubling 1.12; 95% CI 0.73–1.72; *p* = 0.597), nor was operative time (OR per 10 min 1.03; *p* = 0.294). Wound infection was the only variable significantly associated with AL (OR 3.48; 95% CI 1.06–11.44; *p* = 0.042), although its temporal relationship with AL could not be established from the available data. AL rates by experience category were 8.9%, 7.5%, and 7.9% for surgeons with <100, 100–199, and ≥200 cases, respectively (*p* = 0.913). AL was associated with a significantly prolonged hospital stay (median 17 vs. 7 days, *p* < 0.001) regardless of surgical approach. *Conclusions*: Cumulative surgeon experience was not independently associated with AL risk in the selected sample of colorectal resections with primary anastomosis in this single-center, mid-volume setting. Wound infection emerged as the only variable significantly associated with AL, although its temporal relationship with AL could not be determined and several established confounders, including anastomotic height, BMI, ASA class, and emergency status, were unavailable for adjustment. Considerable inter-surgeon variability was observed irrespective of case volume. These findings highlight the complexity of AL risk and the need for prospective multicenter studies with comprehensive risk adjustment.

## 1. Introduction

Anastomotic leakage (AL) after colorectal resection is one of the most feared complications in colorectal surgery, carrying substantial morbidity and mortality [1]. Despite advances in surgical techniques, the incidence of AL has not changed significantly in recent decades and is reported in the literature to vary from 2.8% to as high as 30%, depending on the anastomotic site, operative technique, and institutional factors [1,2]. This variability is partly explained by the lack of a universally accepted definition; the International Study Group of Rectal Cancer (ISREC) definition is currently the most widely recommended universal definition for colorectal AL [1,3]. In many clinical series, AL is diagnosed early, often around postoperative day (POD) 5, though late presentations also occur [4].

A broad range of patient- and procedure-level risk factors for AL have been identified. Meta-analytic evidence points to male sex, elevated BMI, diabetes, coexisting pulmonary disease, higher ASA score, emergency surgery, and open surgical approach as significant risk factors. Among procedural factors, low anastomosis carries a considerably higher risk of dehiscence, and studies have demonstrated reduced leak rates when a defunctioning stoma is used in low anterior resection by diverting the fecal stream [5]. Understanding these risk factors is essential for contextualizing any surgeon-level analysis.

The relationship between surgeon experience and outcomes is typically framed by two paradigms: the learning curve (within-surgeon improvement over time) and volume–outcome (association between case volume and outcomes), which are generally non-linear and may exhibit a ceiling effect [6]. Available data suggest that high-volume surgeons have significantly lower rates of anastomotic leak compared to low-volume surgeons [6,7]. However, the existing literature remains inconclusive on this association, with several large national series failing to demonstrate a significant correlation between surgical volume and anastomotic dehiscence [8,9].

In colorectal surgery, operative time is frequently used as a sensitive indicator of technical progress and learning, and can also be considered a marker of case complexity potentially related to postoperative complications [10]. While CUSUM-based analyses have proposed specific proficiency thresholds for laparoscopic colorectal procedures [11,12,13], no universally accepted cut-off exists, and the present study does not apply threshold-based learning curve analysis; rather, cumulative experience was modeled as a continuous log-transformed variable in the GEE framework.

Most evidence on volume–outcome relationships originates from large national registries or high-volume specialized centers, primarily from Western Europe and North America. Data from mid-volume, non-specialized centers in Central and Eastern Europe remain scarce, limiting the generalizability of existing thresholds to different healthcare settings and case-mix profiles. The present study was conducted in this context. Since cases operated by the same surgeon share unmeasured surgeon-level characteristics, standard regression models that assume independence of observations are not appropriate. A GEE model clustered by surgeon was therefore employed to account for this within-surgeon correlation and provide valid inference in a multi-surgeon setting. We hypothesized that cumulative surgeon experience would not be independently associated with AL risk after adjustment for operative time and other covariates in a mid-volume, multi-surgeon setting.

The aim of this study was to describe the incidence and timing of anastomotic leakage in a single-center cohort of colorectal resections with primary anastomosis performed over a seven-year period (2018–2024), to evaluate the association between cumulative surgeon experience, surgeon age, operative time, and AL risk using a multivariable GEE model clustered by surgeon, to analyze the distribution of AL rates across surgeon experience categories and inter-surgeon variation in outcomes, and to assess the clinical impact of AL on length of hospital stay stratified by surgical approach.

## 2. Materials and Methods

### 2.1. Study Design and Data Source

This was a retrospective observational study of consecutive patients who underwent colorectal resection with primary anastomosis at Békés County Central Hospital, Gyula, Hungary, between January 2018 and December 2024. Data were extracted retrospectively from electronic medical records, including operative reports, discharge summaries, and postoperative follow-up documentation, by a single investigator (R.S.H.) between February and May 2025. As data extraction was performed by a single investigator without independent verification or inter-rater reliability assessment, the possibility of systematic extraction bias cannot be excluded.

### 2.2. Study Population

All consecutive adult patients who underwent colorectal resection with primary anastomosis for either benign or malignant indications during the study period were eligible for inclusion.

**Figure 1:**
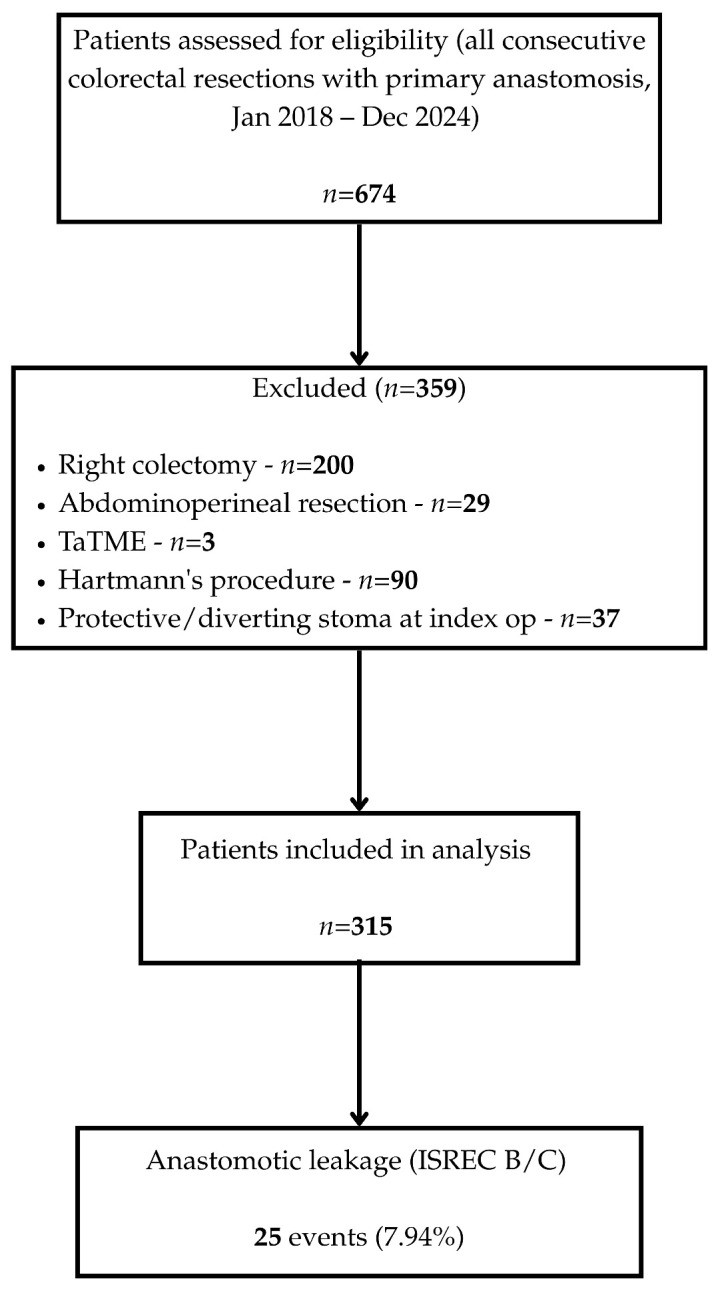
Flow diagram of patient selection.

Patients were excluded if they underwent right colectomy, as ileocolic anastomoses carry a substantially lower and more homogeneous risk of anastomotic leakage compared to left-sided and rectal anastomoses, and their inclusion would introduce outcome heterogeneity that could obscure the association between surgeon experience and AL risk in the segments of primary interest [5]; abdominoperineal resection, which does not involve an anastomosis; transanal total mesorectal excision (TaTME), due to its distinct technique and learning curve; or Hartmann’s procedure. Cases in which a protective stoma was fashioned at the time of the index procedure were also excluded, as the presence of a diverting stoma modifies the clinical expression and consequences of anastomotic leakage (Figure 1). It should be noted, however, that the decision to create a diverting stoma is not random but reflects individual surgeon judgment regarding perceived leakage risk, particularly in the context of low rectal anastomoses or high-risk patient profiles. The implications of this exclusion for the interpretation of surgeon-level comparisons are addressed in the Limitations paragraph, at the end of the Section 4.

### 2.3. Variables

The primary outcome was anastomotic leakage (AL), recorded as a binary variable (yes/no). AL was defined according to the International Study Group of Rectal Cancer (ISREC) criteria as a defect of intestinal wall integrity at the anastomotic site leading to communication between the intra- and extraluminal compartments [3]. Only clinically relevant leaks corresponding to ISREC grade B or C, requiring active intervention such as radiological drainage, endoscopic treatment, or surgical reintervention, were recorded as events. AL was considered present if diagnosed during the index hospitalization or within 90 days of surgery, based on clinical, radiological, or intraoperative findings. For all AL cases, the postoperative day (POD) at diagnosis was recorded.

The main exposure variable was surgeon experience, defined as the cumulative number of colorectal resections with primary anastomosis performed by each surgeon at Békés County Central Hospital, Gyula, as extracted from institutional operative registries up to and including the study period. This definition reflects institution-specific experience only and does not capture experience acquired at prior institutions. Surgeons classified as lower-volume within this dataset may therefore possess substantial prior colorectal surgical experience, introducing potential misclassification of the main exposure variable. The implications of this limitation are discussed further below.

Covariates included age, sex, benign or malignant pathology, resected segment (left hemicolon, transverse colon, sigmoid, rectosigmoid), surgical approach (open or laparoscopic), conversion to open surgery, anastomosis type (handsewn vs. stapled), presence of wound infection, and operative time in minutes. Several variables with established relevance to AL risk were not available for inclusion in the model, including BMI, ASA classification, diabetes status, emergency versus elective presentation, presence of obstruction or perioperative sepsis, nutritional status, steroid use, neoadjuvant treatment, and anastomotic height from the anal verge. The implications of these omissions for model completeness and risk adjustment are addressed in the Limitations section. To protect individual privacy, surgeons were anonymized and coded A–H throughout the analysis.

### 2.4. Statistical Analysis

Continuous variables were tested for normality using the Shapiro–Wilk test and described as mean ± SD or median (IQR) accordingly. Categorical variables were expressed as absolute frequencies and percentages. Univariate comparisons used the Mann–Whitney U test for continuous variables and Fisher’s exact test for categorical variables, applied both to AL vs. no-AL comparisons and to subgroup analyses by surgical approach; for contingency tables larger than 2 × 2, the Fisher–Freeman–Halton extension was used. The association between cumulative surgeon experience (log2-transformed) and operative time was assessed using Spearman’s rank correlation coefficient. Wilson confidence intervals were calculated for individual surgeon anastomotic leakage rates to account for the small number of cases per surgeon.

The main predictor was log2-transformed cumulative experience, which renders the estimated odds ratio (OR) interpretable as the change in AL risk per doubling of cumulative experience. Operative time was entered as a continuous variable per 10 min increment as a covariate, with the aim of adjusting for case complexity. It is acknowledged, however, that operative time may also lie on the causal pathway between surgeon experience and anastomotic leakage, and its inclusion in the model may therefore introduce partial overadjustment. To address this, a pre-specified sensitivity analysis was performed excluding operative time from the GEE model, to assess whether its inclusion materially affected the estimated association between experience and AL risk. In the primary GEE model, cumulative surgeon experience was analyzed as a continuous log2-transformed variable. The categorization of surgeons into experience strata (<100, 100–199, and ≥200 cumulative cases) was not pre-specified and was performed post hoc for descriptive purposes only, in order to present AL rates across clinically interpretable volume bands; these categories were not used for inferential testing of the primary hypothesis, and the corresponding between-group comparison should be regarded as exploratory. Statistical significance was set at α = 0.05. It is acknowledged that the GEE model was estimated with only eight surgeon clusters, which is below the threshold typically recommended for asymptotically valid sandwich variance estimation. In this context, the resulting standard errors and confidence intervals should be interpreted with caution, as they may be underestimated; findings should be considered exploratory rather than confirmatory. In addition, the multivariable model included six covariates for only 25 anastomotic leakage events, yielding an events-per-variable ratio of approximately 4, well below the conventionally recommended minimum of 10. This raises a substantial risk of overfitting and may render the coefficient estimates unstable and prone to bias; the multivariable results should therefore be interpreted with corresponding caution.

No formal a priori sample size calculation was performed, as the study included all eligible consecutive cases operated during the defined study period. Given the relatively low number of AL events (n = 25), the available sample provides limited statistical power to detect modest effect sizes, and findings should be interpreted accordingly. A post hoc exploratory power calculation was therefore performed for the main predictor, cumulative surgeon experience (log2-transformed), based on the observed sample size, event rate, and effect estimate; this calculation was based on a standard logistic framework and did not account for the clustered GEE structure with eight surgeon clusters, and should be regarded as an approximate estimate. A pre-specified sensitivity analysis was performed excluding the single AL case diagnosed beyond 30 days (POD 63), to assess the robustness of the primary findings. The 30-day cut-off was selected in accordance with the conventional distinction between early and late anastomotic leakage; on this basis, the POD 63 case fell outside the early window, whereas the POD 28 case remained within it and was retained.

All statistical analyses were performed using IBM SPSS Statistics (version 25, IBM Corp., Armonk, NY, USA).

## 3. Results

Among 315 patients included, AL occurred in 25 cases, corresponding to an overall incidence of 7.94%. AL was diagnosed at a median of POD 5 (IQR 4.5–8; mean 9.08 ± 12.22 days), with two late presentations at POD 28 and POD 63. The median age was 68 years (IQR 62–74), with a male predominance (61.0%). Most cases involved malignant pathology (82.9%), and 73.3% presented with at least one comorbidity. Rectosigmoid was the most frequently resected segment (49.8%), followed by sigmoid (26.7%), left hemicolon (12.1%), and transverse colon (11.4%). An open approach was used in 58.7% of cases; of the 130 laparoscopic procedures, 24 (18.5%) required conversion. Anastomosis was hand-sewn in 54.0% and stapled in 46.0% of cases. When stratified by anastomotic leakage status, none of the baseline characteristics differed significantly between patients with and without AL (all *p* > 0.05; Table 1).

Eight surgeons participated in the study. By cumulative case volume, two surgeons (25.0%) had performed fewer than 100 colorectal anastomoses, two (25.0%) between 100 and 199 cases, and four (50.0%) had experience of 200 or more cases. Total case volume ranged from 50 to 600 procedures per surgeon. Median operative time for the entire cohort was 100 min (IQR 75–120), ranging from 40 to 240 min. Median postoperative discharge occurred on day 7 (IQR 5–8).

Across the entire sample, surgeon age and operative time did not differ significantly between patients with and without AL (*p* = 0.616 and *p* = 0.742, respectively), while postoperative discharge was significantly later in the AL group (median 17 vs. 7 days, *p* < 0.001). The same pattern was observed in the open surgery subgroup, where neither surgeon age (*p* = 0.864) nor operative time (*p* = 0.901) was associated with AL, but discharge day remained significantly longer in AL cases (median 18 vs. 7 days, *p* < 0.001). In the laparoscopic subgroup, surgeon age was not significantly associated with AL (*p* = 0.624), and operative time did not reach statistical significance, though a numerical difference was observed between AL and non-AL cases (median 135 vs. 110 min, *p* = 0.080), while postoperative discharge was again significantly prolonged in AL cases (median 14 vs. 6 days, *p* < 0.001) (Table 2).

Table 3 shows significant differences between open and laparoscopic approaches across all three variables. Laparoscopic procedures were performed by significantly younger surgeons (median 48 vs. 56 years, *p* < 0.001) and were associated with longer operative time (median 110 vs. 85 min, *p* < 0.001). Patients undergoing laparoscopic surgery were discharged significantly earlier than those in the open group (median 6 vs. 7 days, *p* < 0.001). Cumulative surgeon experience, expressed as log2-transformed case volume, was significantly inversely correlated with operative time (Spearman’s rs = −0.240; 95% CI −0.340 to −0.133; *p* < 0.001), indicating shorter operative duration with increasing surgeon experience.

Table 4 presents the results of the multivariable GEE model for AL, clustered by surgeon. Surgeon experience was not significantly associated with AL risk (OR per doubling 1.12; 95% CI 0.73–1.72; *p* = 0.597), nor was operative time (OR per 10 min 1.03; 95% CI 0.98–1.09; *p* = 0.294); however, the wide confidence interval does not exclude a clinically meaningful association in either direction. Among the covariates included in the model, wound infection was the only variable reaching statistical significance in the multivariable GEE model (OR 3.48; 95% CI 1.06–11.44; *p* = 0.042); however, the temporal relationship between wound infection and anastomotic leakage could not be established from the available data, and this association should be interpreted with caution. Laparoscopic approach, conversion, and rectosigmoid resection were not significantly associated with AL. A post hoc exploratory power calculation for the main predictor indicated that, given 315 patients, 25 anastomotic leakage events, an event rate of 7.94%, α = 0.05, and the observed OR of 1.12, the achieved statistical power was low (approximately 9.1%), with a minimum detectable OR of approximately 1.71 at 80% power.

A post hoc descriptive analysis by experience category (Table 5), using strata defined for reporting purposes only, showed AL rates of 8.9% in surgeons with fewer than 100 cases, 7.5% in the 100–199 group, and 7.9% in those with 200 or more cases, with no statistically significant difference between groups (Fisher–Freeman–Halton exact test, *p* = 0.913). At the individual surgeon level (Table 6), AL rates varied considerably, ranging from 0.0% (Surgeon A, 50 total cases) to 16.0% (Surgeon B, 80 total cases), with no consistent trend by cumulative experience. Among surgeons with ≥200 cases, AL rates ranged from 3.9% to 12.2%, suggesting that between-surgeon variability reflects factors beyond cumulative case volume alone.

Sensitivity analysis was performed by excluding the single AL case diagnosed beyond 30 days (POD 63). The exclusion did not materially change the results: the OR per doubling of surgeon experience was 1.08 (*p* = 0.70) and the OR per 10 min of operative time was 1.03 (*p* = 0.28), confirming the robustness of the primary analysis findings.

A second sensitivity analysis was performed by excluding operative time from the multivariable GEE model, to evaluate the potential for overadjustment arising from its dual role as a marker of case complexity and a possible mediator of the experience–AL relationship. In this reduced model, the OR per doubling of cumulative surgeon experience was 1.11 (95% CI 0.69–1.76; *p* = 0.673), which was not materially different from the primary analysis estimate (OR 1.12; *p* = 0.597), suggesting that the inclusion of operative time did not substantially alter the estimated association between experience and anastomotic leakage risk.

## 4. Discussion

This retrospective study analyzed 315 colorectal resections with primary anastomosis performed between 2018 and 2024, evaluating the association between surgeon experience and the risk of AL in a selected mid-volume cohort with limited risk adjustment. The overall AL incidence was 7.94%, with a predominantly early onset (median POD 5). No significant association was detected between cumulative surgeon experience and AL risk in the multivariable GEE model (OR per doubling 1.12; 95% CI 0.73–1.72; *p* = 0.597); however, the wide confidence interval, spanning from a potential 27% reduction to a 72% increase in AL risk per doubling of experience, precludes definitive conclusions regarding the absence of an effect, and this finding should not be generalized beyond the present setting. Operative time was likewise not an independent predictor in this cohort (OR per 10 min 1.03; 95% CI 0.98–1.09; *p* = 0.294). Within the available covariates, wound infection was the only variable reaching statistical significance (OR 3.48; *p* = 0.042), though this association should be interpreted cautiously given the inability to establish its temporal relationship with AL from the available data. At the descriptive level, AL rates varied considerably among surgeons (0–16%), with no consistent trend in relation to cumulative case volume.

The relationship between surgical volume and postoperative outcomes has been extensively studied in colorectal surgery, described within two main paradigms: the learning curve, which reflects individual performance improvement over time, and the volume–outcome relationship, which associates institutional or individual volume with clinical outcomes [6,14]. Retrospective studies and meta-analyses have demonstrated that higher volumes are generally associated with lower complication rates, although this relationship is non-linear and without a universally accepted threshold [2,15]. In laparoscopic colorectal surgery, CUSUM-based analyses have estimated that technical proficiency may be achieved after approximately 55 cases for right-sided resections and 62 for left-sided resections, while for laparoscopic rectal surgery the reported thresholds range from 50 to 90 cases [11,13]. Operative time is considered one of the most sensitive markers of progression along the learning curve, reflecting the technical efficiency gained with accumulating experience [11], although some studies suggest that this relationship is not always linear, as longer operative times may reflect surgeons’ willingness to undertake more complex cases as their experience grows [16].

The AL incidence in our sample was 7.94%, consistent with data from the literature. Rennie et al. report rates of up to 30% depending on the type of resection, the definition used, and the characteristics of the study population, a variability confirmed by Tsalikidis et al. in a systematic analysis of predictive factors for AL in colorectal surgery [1,17].

The significant prolongation of postoperative hospital stay in the AL group, by approximately 10 days compared to cases without AL, regardless of surgical approach, reflects the substantial clinical impact of this complication. Our results are consistent with data from the literature. Nijssen et al. demonstrated in a systematic review that AL represents a major economic and clinical burden, with additional costs driven primarily by readmissions, prolonged hospital stay, and reinterventions, with incremental costs ranging from €2250 to €83,633 depending on the country [18].

Surgeon age was not significantly associated with AL risk in any of the analyzed subgroups, a result consistent with the observations of García-Granero et al. and Marinello et al., who demonstrated that inter-surgeon variability in AL rates reflects individual-specific factors beyond simple accumulated experience or age [19,20]. Operative time was not a significant predictor at the level of the entire cohort or in the open surgery subgroup; however, in the laparoscopic subgroup a trend toward statistical significance was observed (median 135 vs. 110 min, *p* = 0.080), suggesting that AL cases involved technically more difficult or complex procedures, an aspect documented by Poles et al. in the context of the relationship between operative duration and postoperative complications [21]. The interpretation of surgical approach as a covariate in the present analysis warrants caution. As shown in Table 3, laparoscopic procedures were performed predominantly by younger surgeons and were associated with longer operative times, suggesting that surgical approach is not randomly distributed across surgeons but is intertwined with surgeon profile, experience level, and likely case selection patterns. In this context, surgical approach cannot be treated as a simple technical covariate; it may act as a proxy for surgeon-level characteristics and case complexity, introducing collinearity that limits the independent interpretability of each variable in the multivariable model.

Wound infection was the only significant independent predictor identified in the multivariable model (OR 3.48; *p* = 0.042), an association documented by Tsalikidis et al. and Zarnescu et al., who highlight the role of perioperative inflammatory status in compromising anastomotic integrity [1,17]. However, this association requires cautious interpretation. In a retrospective dataset without systematic recording of the exact postoperative day of wound infection diagnosis relative to the day of AL diagnosis, the temporal sequence between these two events cannot be established with certainty. Wound infection may represent a true antecedent risk factor (for example, through systemic inflammatory burden compromising tissue perfusion and anastomotic integrity), but it may equally represent a concurrent manifestation of the same underlying septic process, a consequence of an evolving anastomotic leak propagating to the wound, or a parallel correlate rather than an independent cause [1,17]. This ambiguity limits the extent to which wound infection can be interpreted as a straightforward independent predictor of AL in the present analysis. The considerable variability in AL rates among individual surgeons, ranging from 0% to 16%, with no consistent trend in relation to cumulative case volume, even in the group with experience ≥200 cases, suggests that factors such as individual technique and case complexity contribute more than cumulative experience alone.

The identification of reliable biomarkers for the early prediction of postoperative complications after colorectal resection has become an active area of research. Inflammatory markers such as C-reactive protein, procalcitonin, and white blood cell count, particularly their postoperative trajectories, have been widely investigated as early indicators of anastomotic leakage and infectious complications, supporting decision-making regarding safe early discharge within enhanced recovery protocols [22]. More recently, attention has turned to novel biomarkers reflecting the systemic inflammatory response. Among these, butyrylcholinesterase (BChE), a non-specific cholinesterase enzyme whose serum levels decline during systemic inflammation and sepsis, has emerged as a promising candidate. In a prospective study of patients undergoing colorectal surgery, lower postoperative BChE levels were significantly associated with both the risk and the severity of surgical site infections, suggesting its potential utility as an early predictive marker [23]. Although such biomarkers were not available in our retrospective dataset, their incorporation into future prospective studies could enhance risk stratification and complement surgeon- and procedure-level factors in predicting anastomotic leakage and related complications.

Surgical site infections (SSIs) are among the most frequent complications after colorectal surgery and are closely linked to anastomotic integrity, as local infection and inflammation may impair healing at the anastomotic site. Colorectal SSIs are typically polymicrobial, with *Escherichia coli* being the most commonly isolated pathogen, followed by *Enterococcus* and *Klebsiella* species [24]. This is consistent with the overlap between wound infection and anastomotic leakage observed in our sample. Postoperative sepsis represents one of the most serious consequences of infectious complications after colorectal surgery and is closely associated with anastomotic leakage and surgical site infection. Prospective observational data indicate that sepsis following colorectal surgery is associated with considerable morbidity, prolonged hospitalization, and increased mortality, underscoring the importance of early recognition and timely intervention [25]. These observations reinforce the clinical relevance of the infectious complications identified in our sample, in which wound infection emerged as the only significant predictor of anastomotic leakage.

Beyond their physical consequences, postoperative complications following colorectal cancer surgery carry a substantial psychological burden. Anxiety and depression are common among colorectal cancer patients, and the occurrence of postoperative complications has been identified as an independent risk factor for postoperative psychological distress, partly mediated by prolonged hospitalization, delayed recovery, and the presence of a stoma [26]. Given that anastomotic leakage and related infectious complications markedly prolong hospital stay, as observed in our cohort, their potential impact on patients’ mental health and quality of life should not be overlooked, reinforcing the importance of integrated psychological support within comprehensive postoperative care.

The robustness of the primary findings was confirmed by sensitivity analysis, in which the exclusion of the late AL case (POD 63) did not materially change the GEE model estimates. Sensitivity analysis is relevant in this context because late AL presentations may reflect distinct pathophysiological mechanisms compared to early dehiscence (including progressive ischemia, chronic infection, or factors related to long-term tissue healing) and could introduce heterogeneity into the model [27]. The stability of estimates after excluding this case reduces the likelihood that atypical presentations influenced the primary conclusions.

The study presents several notable strengths. The use of the GEE model with clustering by surgeon represents a methodologically rigorous approach, accounting for the non-independence of cases operated by the same surgeon, a source of bias frequently overlooked in retrospective studies in colorectal surgery, which can lead to artificially narrow confidence intervals and false-positive conclusions [28]. The inclusion of all consecutive eligible cases over a 7-year period, in accordance with STROBE guidelines for observational studies, reduces the risk of selection bias [29]. The stratified analysis by surgical approach provides a more nuanced perspective on the relationship between the studied variables and AL risk. Surgeon anonymization allowed transparent reporting of inter-individual variability without compromising confidentiality.

The study has several limitations. The retrospective single-center design limits the possibility to generalize the conclusions and exposes the study to information and selection bias inherent to this type of analysis. Surgeon experience was defined exclusively as cumulative case volume at the study institution, which represents a potentially important source of exposure misclassification. Surgeons categorized as less experienced in the institutional registry may in reality have acquired substantial colorectal surgical experience at prior institutions, meaning that the low-experience category in our analysis does not necessarily reflect true surgical inexperience. This form of misclassification is non-differential with respect to the outcome and would be expected to bias the observed association toward the null, potentially obscuring a true relationship between experience and anastomotic leakage risk. Consequently, the absence of a statistically significant association in our GEE model should be interpreted with caution and cannot be taken as definitive evidence that surgeon experience does not influence AL risk. The relatively small number of AL events (n = 25) limits the statistical power to detect moderate effects after multivariable adjustment. A post hoc exploratory power calculation confirmed this, indicating an achieved power of approximately 9.1% for the observed OR of 1.12, with an OR of approximately 1.71 required to reach 80% power. Accordingly, the non-significant association between surgeon experience and anastomotic leakage should not be interpreted as evidence of no effect, but rather as a reflection of limited statistical power to detect small-to-moderate associations. Relatedly, the multivariable GEE model included six covariates for only 25 events, corresponding to an events-per-variable ratio of approximately 4. This falls well below the customary threshold of at least 10 events per variable and introduces a meaningful risk of overfitting, with the potential for unstable and biased effect estimates. This further supports an exploratory interpretation of the multivariable findings. The multivariable model was limited by the absence of several established predictors of AL risk. Among these, anastomotic height from the anal verge is particularly consequential, as low rectal anastomoses carry substantially higher leak rates and their distribution across surgeons could confound the experience–AL association [28]. Beyond anastomotic height, variables including BMI, ASA classification, diabetes status, perioperative inflammatory biomarkers (e.g., white blood cell count, neutrophil count, C-reactive protein), emergency versus elective presentation, presence of obstruction or perioperative sepsis, nutritional status, steroid use, and neoadjuvant treatment were not available in the institutional dataset and could not be included in the model. The absence of these covariates means that the GEE model provides only partial risk adjustment, and residual confounding cannot be excluded. In particular, if surgeons with lower institutional case volumes systematically operated on higher-risk patients (for example, more urgent presentations or more distal anastomoses), the observed null association between experience and AL could reflect residual confounding rather than a true absence of effect [30,31]. These omissions represent a substantive constraint on the interpretability of the primary analysis and should be addressed in future prospective studies with more comprehensive data collection. The sample includes patients with both benign and malignant pathology, multiple resection sites, and both open and laparoscopic approaches, representing substantially different technical and biological risk profiles for anastomotic leakage. Although these variables were included as covariates in the GEE model, the available sample size precluded more granular stratified analyses or interaction modeling, which represents an additional analytical limitation.

The exclusion of all stoma-protected cases deserves more explicit consideration as a potential source of surgeon-dependent selection bias. Protective stomas are not applied randomly; they are typically fashioned in patients judged to be at highest risk of anastomotic leakage, including those with low rectal anastomoses, adverse patient-level risk profiles, or technically demanding procedures [2,32]. By excluding these cases, the study sample is systematically depleted of a clinically important high-risk subgroup. Critically, the threshold for diversion is not uniform across surgeons and varies with individual clinical judgment, experience, and institutional practice patterns [19,20]. Surgeons with a lower threshold for diversion would contribute fewer high-risk cases to the analytic sample, potentially making their observed AL rates appear artificially lower than their true risk-adjusted performance. Conversely, surgeons who rarely divert may retain all high-risk cases in the dataset [33]. This differential case selection could materially distort surgeon-level comparisons of leakage risk and, consequently, bias the estimation of the experience–AL association in ways that are difficult to quantify from the available data.

Future studies should adopt prospective multicenter designs, allowing more rigorous risk adjustment and greater statistical power to detect moderate effects of surgeon experience on AL [34]. The integration of longitudinal RA-CUSUM analyses per surgeon would provide a more accurate characterization of the dynamics of individual performance over time [35]. The systematic inclusion of variables absent from our study would significantly improve AL risk prediction models. Future studies should also consider restricting analyses to more homogeneous surgical subgroups or explicitly modeling interactions between resection site, surgical approach, and surgeon experience to better isolate the independent contribution of each factor to AL risk.

Beyond the intraoperative and surgeon-level factors examined here, the broader burden of colorectal disease underscores the importance of prevention and early detection. Population-based colorectal cancer screening is well established, yet healthcare workers represent a group in whom screening uptake may be suboptimal, owing to irregular working schedules, high occupational demands, and a tendency to postpone their own preventive care. Integrating colorectal cancer screening into occupational health surveillance for healthcare staff has been associated with increased participation and reduced colorectal cancer mortality, supporting its value as a complementary strategy to existing public health programs [36]. Strengthening preventive efforts in this population may help reduce both the incidence and the late presentation of colorectal disease, including advanced cases that ultimately require resection and carry the attendant risk of postoperative complications such as anastomotic leakage.

## 5. Conclusions

In this selected sample of colorectal resections with primary anastomosis, cumulative surgeon experience was not independently associated with AL risk in a multivariable GEE model with partial risk adjustment; however, this finding should be interpreted cautiously given the absence of several established confounders, including anastomotic height, BMI, ASA class, and emergency status. Wound infection was the only significant predictor identified within the available variables. Inter-surgeon variability in AL rates did not follow a consistent trend with case volume, and AL was associated with a clinically meaningful prolongation of hospital stay regardless of surgical approach. These findings underscore the complexity of AL risk in mid-volume settings and the need for prospective multicenter studies with more comprehensive risk adjustment.

## Figures and Tables

**Table 1:** Baseline characteristics stratified by anastomotic leakage status.

Characteristic	Total (*n* = 315)	No AL (*n* = 290)	AL (*n* = 25)	*p*
Sex				
Male	192 (61.0%)	174 (60.0%)	18 (72.0%)	0.289
Female	123 (39.0%)	116 (40.0%)	7 (28.0%)	
Pathology				
Malignant	261 (82.9%)	239 (82.4%)	22 (88.0%)	0.591
Benign	54 (17.1%)	51 (17.6%)	3 (12.0%)	
Comorbidities				
Present	231 (73.3%)	211 (72.8%)	20 (80.0%)	0.637
Absent	84 (26.7%)	79 (27.2%)	5 (20.0%)	
Resected segment				
Rectosigmoid	157 (49.8%)	142 (49.0%)	15 (60.0%)	0.679
Sigmoid	84 (26.7%)	78 (26.9%)	6 (24.0%)	
Left hemicolon	38 (12.1%)	35 (12.1%)	3 (12.0%)	
Transverse	36 (11.4%)	35 (12.1%)	1 (4.0%)	
Surgical approach				
Open	185 (58.7%)	168 (57.9%)	17 (68.0%)	0.400
Laparoscopic	130 (41.3%)	122 (42.1%)	8 (32.0%)	
Anastomosis type				
Hand-sewn	170 (54.0%)	159 (54.8%)	11 (44.0%)	0.306
Stapled	145 (46.0%)	131 (45.2%)	14 (56.0%)	

**Table 2:** Comparison of operative variables by anastomotic leakage status, stratified by surgical approach.

Variable	No AL	AL	*p*
	Median (IQR)	Median (IQR)	
**Entire** **sample**			
Surgeon age (years)	51.5 (45–59)	56 (44.5–60)	0.616
Operative time (min)	95 (75–120)	100 (77.5–130)	0.742
Postoperative discharge (POD)	7 (5–8)	17 (13–20.5)	<0.001
**Open approach**			
Surgeon age (years)	56 (46–61)	57 (44–61.5)	0.864
Operative time (min)	85 (65–110)	85 (67.5–100)	0.901
Postoperative discharge (POD)	7 (7–9)	18 (13–20.5)	<0.001
**Laparoscopic approach**			
Surgeon age (years)	48 (45–53)	49 (45.25–58.5)	0.624
Operative time (min)	110 (90–130)	135 (106.25–175)	0.080
Postoperative discharge (POD)	6 (5–7)	14 (9.5–20.75)	<0.001

**Table 3:** Comparison of operative variables by surgical approach.

Variable	Open	Laparoscopic	*p*
	Median (IQR)	Median (IQR)	
Surgeon age (years)	56 (46–61)	48 (45–54)	<0.001
Operative time (min)	85 (65–110)	110 (90–131.25)	<0.001
Postoperative discharge (POD)	7 (7–10)	6 (5–7)	<0.001

**Table 4:** Multivariable GEE model for anastomotic leakage (clustered by surgeon).

Predictor	OR	95% CI	*p*
Experience (log2, per doubling)	1.12	0.73–1.72	0.597
Operative time (per 10 min)	1.03	0.98–1.09	0.294
Laparoscopic approach (vs. open)	0.61	0.16–2.37	0.461
Conversion (yes vs. no)	1.11	0.29–4.23	0.875
Wound infection (yes vs. no)	3.48	1.06–11.44	0.042
Rectosigmoid resection (vs. others)	1.83	0.87–3.84	0.112

**Table 5:** Anastomotic leakage rate by surgeon experience category.

Experience Group	2018–2024 Cases	AL (n)	AL Rate	Exact *p*
<100 cases	45	4	8.9%	0.913
100–199 cases	80	6	7.5%	
≥200 cases	190	15	7.9%	

**Table 6:** Anastomotic leakage rate by surgeon (coded A–H).

Surgeon	Total Experience (Cases)	Experience Category	2018–2024 Cases	AL (n)	AL Rate	95% CI (Wilson)
A	50	<100	20	0	0.0%	0.0–16.8%
B	80	<100	25	4	16.0%	4.5–35.1%
C	100	100–199	28	2	7.1%	0.9–23.5%
D	130	100–199	52	4	7.7%	2.1–18.5%
E	200	≥200	51	2	3.9%	0.5–13.5%
F	400	≥200	50	5	10.0%	3.3–21.8%
G	400	≥200	49	6	12.2%	4.6–24.8%
H	600	≥200	40	2	5.0%	0.6–16.9%

## Data Availability

The data presented in this study are not publicly available due to privacy and ethical restrictions. The data are held by Békés County Central Hospital, Gyula, Hungary, and may be made available upon reasonable request to the corresponding author, subject to institutional approval.
